# Type of Cleft and Socioeconomic Determinants for Increased Caries Risk Among Young Patients With Cleft Lip and/or Palate

**DOI:** 10.1177/10556656241299194

**Published:** 2024-11-18

**Authors:** L.S. van der Knaap-Kind, E.B. Wolvius, L. Kragt

**Affiliations:** 1 Department of Oral and Maxillofacial Surgery, Special Dental Care and Orthodontics, Erasmus University Medical Center, Rotterdam, The Netherlands

**Keywords:** dental caries, risk assessment, cleft lip and/or palate

## Abstract

**Objective:**

This study aimed to identify the predictive role of cleft type, ethnicity, adoption status, spoken language at home and parental education level on the caries risk in the primary dentition of patients with cleft lip and/or palate (CL/P). This knowledge is used to make an estimate on increased caries risk in young patients with CL/P.

**Design:**

A retrospective analysis of data concerning dental caries and basic characteristics of patients with CL/P was done. Patients were born and registered in 2016, 2017, or 2018 at the cleft team of the Erasmus Medical Center, Rotterdam, the Netherlands.

**Results:**

After Chi-square tests, the cleft type (*P *= .02), country of birth father (*P *< .001), country of birth mother (*P *= .002), parental educational level (*P *= .006), and spoken language at home (*P *= .002) were significantly different between 144 patients with CL/P with and without caries. Items were used in binary logistic regressions and after stepwise backward elimination resulting in most important determinants for caries in the primary dentition in patients with CL/P being: father born in another country than the Netherlands (odds ratio [OR]* *= 4.87, *P *= .001), a cleft lip alveolus and palate phenotype (OR* *= 3.54, *P *= .002), and a lower parental educational level (OR* *= 2.30, *P *= .04).

**Conclusion:**

The recommendation for the dental care professional will be to use these 3 determinants as a first prediction on future dental caries. This helps the dental professional in clinical decisions as recall intervals, referral to specialized dental care and extensiveness of caries prevention strategies and thereby improves oral health of patients born with CL/P.

## Introduction

Dental caries is the most prevalent noncommunicable disease among children, with a prevalence up to 90% worldwide.^1^ While dental caries occurs frequently among the general population, there are suggestions that children with congenital craniofacial deformities are more often affected.^2^ Cleft lip and/or palate (CL/P) is the most common congenital craniofacial deformity,^3^ but the determinants for dental caries among this patient group are still unclear.

Worth et al^4^ showed that all studies in nonfluoridated areas reported that individuals with CL/P have a greater caries experience than noncleft controls. Although the majority of studies have concluded that individuals with CL/P exhibit a higher caries prevalence compared to those without CL/P, a substantial body of conflicting evidence still exists.^4^ Possible factors that led to inconclusive results are neglecting the type of cleft or neglecting orthodontic appliances. Several factors may contribute to a higher caries susceptibility in patients with CL/P, such as impaired oral hygiene as a result of a reluctance to brush around the cleft area, poorly aligned maxillary dentition and limited access following surgical repair of the upper lip and possible scarring.^5^ Other caries risk factors are enamel hypoplasia,^6^ frequent intake of comfort food like sweets or sugar-containing beverages and early colonization of caries-associated microorganisms.^7,8^ Furthermore, the prolonged oral clearance time in children with CL/P may also contribute to a cariogenic environment.^9^

In the general population, one of the major risk factors for dental caries is a low socioeconomic position (SEP).^10^ Even in a healthcare system where pediatric dental costs are fully covered, caries experience is highly influenced by social factors.^11^ Literature on how SEP influences caries prevalence in patients with CL/P, especially in relation to other potential clinical factors, is limited. CL/P occurs in all socioeconomic levels, but is slightly more prevalent in low SEP.^12^

To provide the best possible dental care it is essential to understand which patients born with CL/P are more susceptible to developing caries already at a young age, so that recall intervals, prevention strategies and referral to specialized dental care can be adapted early conform the caries risk. The aim of the present study is to understand the relative role of cleft type and several SEP factors as ethnicity, adoption status, spoken language at home and parental education level, on the caries experience in the primary dentition in patients with CL/P. This knowledge can be used to estimate increased caries risk for young patients with CL/P.

## Material and Methods

Data for this retrospective study were collected from patient records. The protocol of the current study was reviewed and approved by the Medical Ethical Committee of the Erasmus Medical Centre in Rotterdam (METC-2019-0535). Data used in the present study were anonymized prior to the analysis.

### Study Group

The study group consists of patients with CL/P born in 2016, 2017, or 2018 and treated in the cleft team of the Erasmus Medical Center (Erasmus MC), Rotterdam, The Netherlands. In general, every patient with CL/P in the Netherlands is being treated in 1 of the 8 cleft teams in the country. The Erasmus MC cleft team is one of the biggest teams in the Netherlands in number of patients, with 50 to 60 new patients per year.

### Measures and Data Collection Procedure

Data collection took place within the International Consortium for Health Outcomes Measurement (ICHOM) initiative at the Erasmus MC. This cleft team is collecting and documenting data in a structured manner via the ICHOM Standard Cleft Set (ICHOMSCS) since November 2015 onwards. Within the ICHOMSCS, several data are collected during visits of patients with CL/P at different time-points. In the current study, the following data are analyzed related to SEP-indicators, cofactors and dental caries, collected via ICHOMSCS.

**The following SEP-indicators were obtained from the patient file/ICHOMSCS:**

**Consanguinity:** Children were categorized as consanguine, when both parents were descended from the same ancestor.

**Country of birth child, father, and mother:** Three separate variables were included considering the country of birth from father, mother, and child. For the analysis, the country of birth was categorized as either the Netherlands, or any other country.

**Parental educational level:** Maternal or paternal educational level was categorized based on a modification of the Dutch Standard Classification of Education into 2 levels: lower (no education, primary school, secondary school, and intermediate vocational training) and higher (higher vocational training and university). If educational level of parents differed, the highest level was taken for analysis.

**Spoken language at home:** The variable about the spoken language at home was categorized in either Dutch (the predominant language in the Netherlands) or any other spoken language. In the case of bilingual or multilingual families, parents were asked to choose the language most frequently spoken at home.

**The following cofactors were obtained from the patient file/ICHOMSCS:**

**Adoption:** In case of an adoption, the age of adoption and the country of origin was noted.

**Birth weight:** Birth weight was categorized into low (LBW) (< 2500 g), normal (NBW) (2500-4000 g), and high birth weight (HBW) (≥ 4000 g).

**Comorbidities:** Syndromes, chromosome abnormalities, autism spectrum disorder, organ failure, and Pierre Robin (PR) were included as comorbidities.

**Cleft type:** Four cleft types are differentiated: cleft lip (CL), cleft lip and alveolus (CLA), cleft lip and palate (CLAP), and cleft palate (CP).

**Gender:** Gender of the patient was specified as either male or female.

**Gestational age at birth:** Preterm birth was defined as birth before 37 weeks of pregnancy. Born after 37 weeks of pregnancy was categorized as a term.

**Uni/bilateral:** The cleft types CL, CLA, and CLAP can occur uni- or bilaterally, this was noted.

## Dental Caries

Dental caries was assessed with the decayed, missing and filled teeth index (DMFT). The DMFT is a clinician-reported tool, used in epidemiological studies for the quantitative evaluation of caries experience. For this index, the researcher or clinician determines for each patient the number of teeth (T* *= teeth) with an active caries lesion (D = decayed), those that are missing due to caries (M = missing), and those that have restorations due to caries (F = filled). Small letters are used for the primary dentition (dmft) and sum score dmft ranges from 0 to 20, with a higher score for a higher caries experience. The dmft scores are standard collected in the ICHOMSCS at age 5 (within the range of 3 years-11 months and 6 years-1 month) and are scored after intra-oral inspection by a pediatric dentist, a maxillofacial surgeon or an orthodontist, or in rare cases after inspection of the intra-oral pictures by a pediatric dentist. According to the Erasmus MC treatment protocol, patients with CL/P in this age-group are not yet wearing orthodontic appliances.

To test for reliability of the dmft scoring during the cleft team consultation hours, the intra-oral photographs of 20 randomly chosen patients with CL/P were scored by a pediatric dentist (LSKK), resulting in a very good reliability of κ=0.841.

### Data Analysis

IBM SPSS statistics version 28 was used for statistical analysis (IBM Corp., Armonk, NY, USA). Descriptive statistics were used to describe the study population. The mean dmft scores are calculated and presented as mean values with standard deviations (sd) per cleft type. One sample t-test compared mean dmft score of the CL/P group with mean dmft score obtained from van Meijeren-van Lunteren et al.^13^ In this study, the mean dmft value was determined for 4146 children aged 6 years, within a large-scale population-based cohort in the general population, conducted in the same geographical area as the current study.^13^

Differences in the categories of all SEP indicators and cofactors were assessed for patients with CL/P with and without caries experience with Chi-square tests, the dmft index was then dichotomized into dmft = 0 versus dmft >0.

A prediction model was developed by performing binary logistic regression analysis. To prevent overfitting of the prediction model, only candidate predictors that showed an association with presence of dental caries were selected for the prediction modeling, with a cut-off *P*-value of .20. Dental caries was entered as dependent variable, while candidate predictors—determined with the univariate analysis—were entered as factors. The factors were dichotomized for the analysis. Stepwise backward elimination by likelihood ratio removed the predicting items with the highest *P*-value from the model step by step. Odds ratios and *P*-values were reported and a *P-*value < .05 was considered significant. Finally, a power calculation was done, using the Multiple Regression Sample Size Calculator.^14^

## Results

Initially, the 168 patients born and registered at the cleft team Erasmus MC in the years 2016, 2017, and 2018 were included in the study sample. However, for 10 patients no thorough intra-oral examination was possible due to lack of cooperation, another 14 patients moved to another cleft team and were lost to follow-up, meaning that a total of 24 patients had no dmft data available. Therefore, the study sample finally consisted of 144 patients. Table 1 shows the characteristics of the 144 included patients. The majority of the study population and their parents (98 fathers, 101 mothers, and 129 children) were born in the Netherlands whereas 46 fathers, 43 mothers, and 15 children were born somewhere else. Next to the Netherlands, there was a total of 25 different ethnicities.

**Table 1.** Characteristics Considering SEP Indicators and Cofactors of the Study Population Aged Around 5 Years (N = 144), in Alphabetic Order, With and Without Caries Experience in Numbers and Percentages.

		dmft = 0 (N = 78)	dmft > 0 (N = 66)	*P-*value
**Adoption**	Yes	4 (5)	2 (3)	.53
	No	74 (95)	64 (97)	
**Birth weight**	Low	10 (13)	10 (15)	.91
	Normal	56 (72)	47 (71)	
	High	8 (10)	6 (9)	
	Missing	4 (5)	3 (5)	
**Cleft type**	CL	6 (8)	4 (6)	.02 *
	CLA	15 (19)	6 (9)	
	CLAP	22 (28)	35 (53)	
	CP	35 (45)	21 (32)	
**Comorbidities**	Yes	11 (14)	6 (9)	.39
	No	67 (86)	58 (88)	
	Missing	0 (0)	2 (3)	
**Consanguinity**	Yes	2 (3)	5 (8)	.17
	No	72 (92)	59 (89)	
	Missing	4 (5)	2 (3)	
**Country of birth child**	The Netherlands	72 (92)	57 (86)	.25
	other	6 (8)	9 (14)	
**Country of birth father**	The Netherlands	63 (81)	35 (53)	<.001 *
	other	15 (19)	31 (47)	
**Country of birth mother**	The Netherlands	63 (81)	38 (58)	.002 *
	Other	15 (19)	28 (42)	
**Educational level (parental)**	Lower	25 (32)	36 (55)	.006 *
	Higher	43 (55)	23 (35)	
	Missing	10 (13)	7 (11)	
**Gender**	Male	47 (60)	44 (67)	.43
	Female	31 (40)	22 (33)	
**Gestational age at birth**	Preterm	9 (12)	8 (12)	.93
	A term	65 (83)	55 (83)	
	Missing	4 (5)	3 (5)	
**Uni/bilateral**	Unilateral	34 (44)	36 (55)	.92
	Bilateral	9 (12)	9 (14)	
	N.A. (CP)	35 (45)	21 (32)	
**Spoken language at home**	Dutch	71 (91)	47 (71)	.002 *
	Other	7 (9)	19 (29)	

*P*-value < .05 was considered significant.

*Significant difference based on Chi-square tests.

Abbreviations: CL, cleft lip; CLA, cleft lip and alveolus; CLAP, cleft lip and palate; CP, cleft palate; dmft, decayed, missing, filled teeth; N.A., not applicable; SEP, socioeconomic position.

In the current study population, 78 patients had no caries (dmft = 0), 34 patients had mild caries (dmft 1-3) and 32 patients had severe caries (dmft > 3). The mean dmft was 2.08 (3.41). The mean dmft (sd) per cleft type is CL = 0.80 (1.14), CLA = 1.67 (3.47), CLAP = 2.77 (3.89), and CP = 1.75 (3.04). Mean dmft of the population born with CL/P (2.08 [3.41]) was significantly higher than the mean dmft of the general population (0.97 [2.17]), (t[143]* *= 3.90, *P *< .001).

Six patients were adopted, all 6 were adopted from China. The children were adopted at the age of 1, 2 (2x), 4 (2x), and 5 years.

Based on the univariate analysis, the final prediction model for dental caries in patients with CL/P included the following 6 indicators (the characteristics with *P *< .20 as presented in Table 1): cleft type (χ^2^ [3144] = 9.79, *P *= .02), consanguinity (χ^2^ [1136] = 1.86, *P *= .17), country of birth father (χ^2^ [1144] = 12.65, *P *< .001), country of birth mother (χ^2^ [1144] = 9.18, *P *= .002), educational level (χ^2^ [1127] = 7.44, *P *= .006), and spoken language at home (χ^2^ [1144] = 9.49, *P *= .002). These SEP indicators/cofactors were used as the factors in the binary logistic regression.

The binary logistic regression output revealed after stepwise backward elimination a significant coefficient for father's country of birth other than the Netherlands (*P *= .001). Specifically, this coefficient translates to an odds ratio (OR) of 4.87, meaning that having a father who is born abroad increases the likelihood of developing caries with almost 5 times. Furthermore, the binary logistic regression output revealed a significant coefficient for cleft type CLAP (*P *= .002), with an OR of 3.54. Finally, the binary logistic regression output revealed a significant coefficient for the lower educational level (*P *= .043). This coefficient translates to an OR of 2.30, as presented in Table 2.

**Table 2.** Most Relevant Predictors of Dental Caries in the Primary Dentition of the CL/P Population Based on Binary Logistic Regression After Stepwise Backward Elimination.

	n/N	95% CI for odds ratio			*P*-value
		Odds ratio	Lower	Upper	
**Country of birth father other than the Netherlands**	31/46	4.58	1.89	12.59	.001*
**Patient cleft type CLAP**	35/57	3.58	1.57	8.01	.002*
**Lower parental educational level**	36/61	2.30	0.95	4.65	.043*

R^2^, 0.20 (Cox-Snell), 0.27 (Nagelkerke).

*P*-value < .05 was considered significant.

*Significant coefficient based on binary logistic regression.

Abbreviations: CLAP, cleft lip and palate; n, number of persons with caries; N, sum of persons with and without caries.

To detect an effect size of f^2 ^= 0.15, with a significance level of α = 0.05, a statistical power of β = 0.8, and 6 predictor variables, the minimum required sample size is calculated to be 97.^14^

## Discussion

Patients with CL/P experience more dental caries than nonaffected peers, requiring increased access to dental prevention and care.^13^ The aim of the present study was to understand the individual risk of patients with CL/P for future dental caries experience. Based on this study the most extensive cleft phenotype, that is, CLAP, a lower parental educational level and having a father born abroad contributed the most to the risk of developing dental caries in the primary dentition in patients born with CL/P. Adhering these results, the cleft team of Erasmus MC screens on these items during the team visit at age 1 year.

In patients with CL/P who experience more dental fear and often have lower coping behavior in the dental setting due to a higher level of exposure to medical interventions at a young age, caries prevention is very important.^15^ Dental clinicians continuously need to balance the benefits and costs of their (preventive) treatment strategy. Due to the multifactorial character of dental caries lesions, it is difficult to predict individual risk. Determinants of increased caries risk help to estimate recall periods, need for referral to specialized dental care and extensiveness of preventive strategies. Subsequently, early caries prediction enhances cost-efficient, value based healthcare and optimal use of limited resources.^16^

An important requirement for predicting an increased caries risk is that it is easy and brings no extra burden to the patient. Caries prediction tools that have been developed for the general population often require extra effort from patient and caregiver and are time-consuming, which is considered suboptimal for patients with CL/P as they already have many medical visits and subsequent burden.^17^ Therefore, the information that needs to be collected for targeted risk assessment should be as limited as possible, should not be considered too sensitive and be available in early life. SEP indicators are strongly related to oral health outcomes, however many of these SEP indicators are not applicable for clinical risk assessment. For example, household income is for many parents a barrier to report, and converting the zip-code into a socioeconomic score is unwieldy and dynamic. An advantage of the indicators used in the current study, is that they are all standardly available from ICHOMSCS.

In previous research it was found that a carious dentition in patients with CL/P was significantly determined by ethnicity (*P* = .002) and deprivation (*P* = .012).^18^ Further, Al Dajani performed a study in 2009 with siblings aged 12 to 29 years, one without and one with CL/P. Al Dajani found significantly more carious lesions in the CL/P sibling and he concluded that subjects with CL/P are more prone to dental caries independently of their socioeconomic position.^19^ Also, in the current study it appears that the cleft type is an important determinant for caries in the primary dentition, next to specific SEP indicators.

One major limitation of the present study is the limited number of included patients and subsequently the large confidence intervals. Moreover, the Cox-Snell = 0.20 and Nagelkerke = 0.27 indicate only a moderate fit of the model. It is likely that there are unmeasured factors, like diet, oral hygiene habits and fluoride use that would explain more variation in the dental caries risk. These primary etiological factors will vary considerably during the first years of patient’ s life and were therefore not included in the current study. Another limitation is the lack of information on food intake, for example the need to use feeding tubes. This study should be repeated in a few years when more data is available from more patients on dental caries, SEP indicators and cofactors, collected via the ICHOMSCS. A multicenter design can help to further increase the number of included patients and thereby increase validity of the study. By repeating this study in several years, determinants of increased risk for dental caries in the permanent dentition may also be identified.

In conclusion, increased awareness of the elevated caries risk in patients with CLAP with father born abroad, and/or parents with a lower educational level is recommended. Implementing additional preventive measures, such as shorter recall intervals or referral to specialized dental care, may be advised to protect the oral health of the most susceptible patients.
